# Supplementary material for: Utility and precision evidence of technology in the treatment of type 1 diabetes: a systematic review
Source: Commun Med (Lond). 2023 Oct 5;3:132. doi: 10.1038/s43856-023-00358-x (PMC10550996; doi:10.1038/s43856-023-00358-x)
Supplement: Supplementary file 1 — Supplementary Information [file 43856_2023_358_MOESM1_ESM.pdf]

Supplementary Table 1: Original type 1 diabetes treatment topics and literature search.

**Topic**	**Total # of Abstracts screened**
Adjunctive therapies	443
Behavioral health	1665
Beta Cell Replacement	528
Exercise	550
Glycemic Targets	3269
Intensive Insulin therapy	1181
Nutrition	699
Transition of Care	112
Diabetes Technology	835

Supplementary Table 2: Search strategy – Medical Subject Headings (MeSH) and free-text terms.

#1	"Diabetes Mellitus, Type 1"[Mesh] OR insulin-dependent diabetes[Title/Abstract] OR “type-one diabetes” OR “type one diabetes” OR “type-1 diabetes” OR “type 1 diabetes” OR “type I diabetes” OR “IDDM” OR “T1DM” OR “T1D” OR “type-1a diabetes” OR "type 1a diabetes" OR “type 1.5 diabetes” OR “double diabetes” OR "autoimmune diabetes" OR "auto-immune diabetes" OR "immune-mediated diabetes" OR "insulin-deficient diabetes" OR "insulin deficient diabetes" OR "insulin-treated diabetes" OR "insulin treated diabetes" OR insulin depend* OR insulin-depend* OR “Ketosis-Prone” AND diabet* OR “Latent Autoimmune Diabetes in Adults” OR “LADA” OR “juvenile onset diabetes” OR "fulminant diabetes"
#2	“treatments” OR “treatment” OR “therapies” OR “therapy” OR model* OR prognosti* OR "survival analys*” OR “outcome” OR “progression” OR “efficacy” OR “effectiveness” OR “longitudinal” OR "time series" or “randomized controlled trial” OR “RCT”
#3	#1 and #2
#4	“continuous subcutaneous insulin infusion pump” OR “insulin infusion pump” OR “insulin pump” OR “pump” OR “CSII” OR “Sensor-augmented pump” OR “sensor augmented pump” OR “SAP” OR “Patch insulin pump” OR “Tubeless Pump” OR “Low Glucose Suspend” OR “LGS” OR Predictive Low Glucose Suspend” OR “PLGS” OR “Automated Insulin Delivery” OR “Automated Insulin Dosing” OR “AID” OR “Closed loop insulin” OR “Closed-Loop insulin” OR “Closed loop” OR “Hybrid Closed Loop” OR “Hybrid Closed-Loop” OR “HCL” OR “Artificial pancreas” OR “AP” OR “Non-FDA approved pumps” OR “Do-it-yourself” OR “DIY” OR “Do it yourself” OR “smart pump” OR “implantable pump” OR “implanted pump” OR “peritoneal delivery”
#5	“continuous glucose monitor” OR “continuous glucose monitoring” OR “CGM” OR “sensor” OR “intermittently scanned CGM” OR “isCGM” OR “real-time continuous glucose monitor” OR “real time continuous glucose monitor” OR “real-time CGM” OR “real time CGM” OR “rtCGM” OR “integrated continuous glucose monitor” OR “iCGM” OR “implanted continuous glucose monitor” OR “implanted CGM”
#6	#3 and #4 and #5
Filters	Age: Children, Adolescent, Elderly, Adult<br>TIME: 2011-2022<br>Type of article: Review, RCT, Cohort, Trial<br>Other: Human, English, Clinical

Supplementary Table 3: Inclusion/Exclusion Criteria for Evidence to Support Recommendations.

**Included:**	**Excluded:**
Evidence-level (EL) 1: Randomized controlled trials (RCTs)<br>Human participants<br>English<br>Published, full article in peer-reviewed journal<br>Published January 1, 2012 or later<br>All persons with type 1 diabetes mellitus<br>All persons with LADA<br>Continuous glucose monitoring (CGM), including<br>Real-time CGM<br>Intermittently scanned CGM<br>Integrated CGM<br>Insulin pump therapy, including<br>Continuous subcutaneous insulin infusion<br>Sensor-augmented insulin pump therapy<br>Patch insulin pumps<br>Closed-loop insulin delivery<br>Automated insulin dosing systems<br>Non-FDA-approved/DIY pumps	EL 1 systematic reviews or meta-analyses of RCTs<br>EL 2 studies: meta-analyses including nonrandomized trials or observational studies; controlled trials without randomization; cohort, case-control, cross-sectional studies; epidemiological studies (including surveys and registry data); open-label extension studies; post-hoc analyses.<br>EL 3 network analyses<br>EL 4 consensus/position/policy statements and guidelines<br>EL 3 studies: case reports/series, preclinical/feasibility/protocol/pilot studies, studies with hypothetical cohorts, basic research<br>EL 4 studies: editorials/letters, opinions, reviews, theory<br>Abstracts only<br>N<50<br>Animal studies<br>Non-English<br>Studies published before year 2012, except when cited as background<br>Studies that focus on diabetes technology or treatments that are no longer relevant to practice at the time of publication<br>Studies with a focus on accuracy of a product/device<br>Persons without diabetes<br>Persons with CFRD, type 2 diabetes, gestational diabetes, diabetes in pregnancy, steroid-induced diabetes, monogenic diabetes, MODY, neonatal diabetes<br>Persons with Diabetes s/p chemotherapy, pancreatectomy, post-transplant, medication/drug-induced

Supplementary Table 4: A priori subgroups proposed for precision medicine analysis in PROPSERO protocol.

Proposed Subgroups for analysis
- Age (including pediatric and geriatric)<br>- Sex<br>- Race/ethnicity<br>- HbA1c<br>- C-peptide<br>- Hypoglycemia unawareness<br>- Body mass index<br>- Duration of diabetes<br>- Age at onset<br>- Complications of diabetes (including end-stage renal disease, vision loss, cardiovascular disease, neuropathy)<br>- Co-existing significant health problems (including cancer/terminal disease)<br>- Co-existing auto-immune diseases (including thyroid, coeliac disease)<br>- Cognitive impairment<br>- Social determinants of health (including insurance coverage, socioeconomic status)<br>- Education (including health literacy/numeracy)<br>- Family structure/support systems
